# A randomized, double-blind, placebo-controlled, multicenter clinical trial for efficacy and safety of traditional Chinese medicine combined with antibiotics in the treatment of bacterial pneumonia in children

**DOI:** 10.1097/MD.0000000000023217

**Published:** 2020-12-11

**Authors:** Lina Wei, Yina Guo, Yutong Fei, Lin Luo, Caiwen Wang, Xuejiao Wang, Haihang Sun, Liang Liu, Jing Han, Xiaoting Ren, Bo Yao, Lei Wang, Yijie Wang, Liping Sun

**Affiliations:** a First Affiliated Hospital to Changchun University of Chinese Medicine, Jilin; b Changchun University of Chinese Medicine, Changchun; c Beijing University of Chinese Medicine; d Research Institute of China Academy of Chinese Medical Sciences, Beijing, China.

**Keywords:** children, community-acquired bacterial pneumonia, herbal medicine, Maxing Shigan Decoction, Qingfei Decoction, randomized controlled trial

## Abstract

**Background:**

Pneumonia is the second leading cause of death in children worldwide after preterm birth and certification. Bacteria, viruses, mycoplasma, and other microorganisms are known to be the main causes of pneumonia, of which bacterial pathogenic factors account for 12.5% of cases. The invention and application of antibiotics have improved the prognosis of children with community-acquired bacterial pneumonia (CABP) to a certain extent, but with the emergence of antibiotic resistance worldwide, the mortality of children with CABP is still high. “Maxing Shigan Decoction” and “Qingfei Decoction” have significant efficacy in the treatment of CABP in children, but there is no standardized randomized controlled trial to systematically evaluate the outcomes.

**Methods:**

This study is a randomized, double-blind, placebo-controlled, multicenter clinical trial that will randomize 240 patients with CABP to group of Oral Maxing Shigan Decoction, group of Qingfei Decoction or group of placebos administered 3 times a day for 7 days. This study will observe a wide range of clinically relevant endpoints that have been used in clinical trials of pneumonia, including but not limited to clinical cure rate, antibiotic application days, complete antipyretic rate, complete antipyretic days, disease efficacy, traditional Chinese medicine syndrome effect, and antibiotic upgrade treatment rates. Safety will be assessed by monitoring for the incidence of adverse events during the study.

**Discussion:**

This clinical trial is the first to evaluate the efficacy and safety of “Maxing Shigan Decoction” and “Qingfei Decoction” in the treatment of children with CABP. The research results will provide a reference for future research design.

**Trial registration:**

Chinese Clinical Trial Registry, ChiCTR1900025354. Registered on 14th October 2019-Retrospectively registered, http://www.chictr.org.cn/.

## 1 Introduction

Pneumonia is a serious global health problem^[1]^ and the most common cause of death worldwide from infectious diseases, which can cause 3.5 million deaths each year.^[2]^ Children, due to their special physiological and pathological factors, have the deadliest cases of pneumonia.^[3]^ In 2017 alone, 808,694 children under the age of 5 died of infectious pneumonia, accounting for 15% of the total deaths due to infectious causes in children under the age of 5.^[4]^ Pneumonia is also the second leading cause of death for children after complications of preterm delivery.^[5]^ Bacteria, viruses, mycoplasma, and other microorganisms are known to be the main causes of pneumonia,^[6]^ of which bacterial pathogenic factors account for 12.5% of cases.^[7]^ At this stage, antibiotics are the most important means of treating bacterial pneumonia and have been used in various bacterial diseases, including humans and animals. The Global Antimicrobial Resistance and Use Surveillance System (GLASS) report released by the World Health Organization in May 2020 reveals a worrying reality: The emergence and spread of antibiotic resistance worldwide has directly led to longer treatment cycles, increased complications, and even death.^[8]^ Currently, 700,000 people die of antibiotic resistance each year, and as the situation continues to deteriorate, it is expected that 10 million people will die of antibiotic resistance in 2050, resulting in economic losses of 100 trillion US dollars.^[9]^ Therefore, it is necessary to control the further spread of bacterial resistance and explore new antibacterial methods.

Chinese medicine is the most important component of complementary and alternative medicine. The use of Chinese herbal medicines to treat infectious diseases has been the experience of the Chinese for thousands of years and is highly effective but only widely employed in China. Therefore, to promote the use of traditional Chinese medicineg for the treatment of infectious diseases worldwide, the large amount of scientific evidences are still needed to prove its effectiveness, especially the evidences coming from randomized, double-blind, placebo-controlled, multicenter clinical trials. In our study, 2 representative prescriptions, Maxing Shigan Decoction and Qingfei Decoction, were used as therapeutic drugs in combination with antibiotics to treat bacterial infectious pneumonia in children. Maxing Shigan Decoction, which is documented in Treatise on Febrile Diseases, contains 4 Chinese traditional medicine plants, including ephedra (Mahuang), bitter apricot seed (Ku Xingren), gypsum (Shigao), and liquorice root (Gancao). It has been widely used in the clinic for the treatment of colds, upper respiratory tract infections, acute bronchitis, pneumoniaand bronchial asthma^[10]^; it has also been proven to have the effect of anti-influenza virus activity^[11,12]^ and ameliorating microvascular hyperpermeability and inflammatory reactions.^[13,14]^ Qingfei Decoction is a prescription created by Professor Wang Lie who is a master of Chinese national medicine. It bases on his 30 years of experience and is used to treat pneumonia; the extract includes baical skullcap root (Huangqin), weeping forsythia capsule (Lianqiao), blackberry lily rhizome (Shegan) and tendrilleaf fritillary bulb (Chuan Beimu). All the information for this study can be obtained from the Chinese Clinical Trial Registry (Registration number: ChiCTR1900025354).

The primary objective of this trial is to evaluate the clinical efficacy of Maxing Shigan Decoction/Qingfei Decoction combined with antibiotics in the treatment of bacterial pneumonia in children. We evaluated the Primary outcome: Clinical cure rate, Antibiotic application days, Secondary outcomes: Complete antipyretic rate, Complete antipyretic days, Achieved the rate of transfer to the ICU indication, disease efficacy, TCM syndrome effect, effect time of antipyretic, cough, cough phlegm, antiasthmatic, effect time of antipyretic, cough, cough phlegm, antiasthmatic, antibiotics upgrade treatment rates. A further important issue is to observe the effect of Maxing Shigan Decoction and Qingfei Decoction on the use of antibiotics in the treatment of children with bacterial pneumonia by evaluating the number of days of antibiotic application and the incidence of antibiotic escalation treatment. Moreover, as this is a clinical trial in children, we will assess safety aspects by comprehensively documenting adverse events.

## 2 Methods and design

### 2.1 Study design

**Figure 1:**
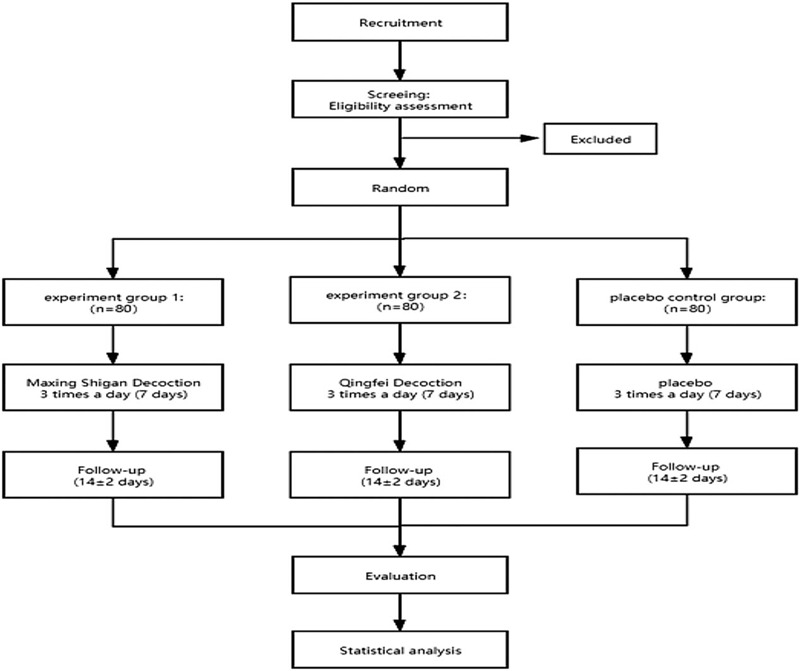
Study flowchart describing the details of the randomized controlled trial. The entire trial includes assessments on 3day, 5day, 7day, and 14 ± 2 days (follow-up visit).

This study is a randomized, double-blind, placebo-controlled, multicenter clinical trial to compare the efficacy and safety of Qingfei Decoction and Maxing Shigan Decoction. It will recruit children with CABP in hospitals. Participants will be randomized to receive Maxing Shigan Decoction, Qingfei Decoction or placebo according to 1:1:1 ratio. Participants will be observed at their hospital admission and outpatient visits after 14 ± 2 days. Specific schedules and investigations are summarized in Table 1 and Figure 1.

**Table 1:** Schedule of enrolment, interventions, and assessments.

	Study period								
Visit	Screening	Treatment observation period							Follow-up
Time point, day	0–1 days	Day 1	Day 2	Day 3	Day 4	Day 5	Day 6	Day 7	14 ± 2
Enrollment:										
Medical history (past, present)	X								
Eligibility screening	X								
Informed consent	X								
Random	X								
Interventions:									
Maxing Shigan Decoction					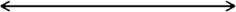				
Qingfei Decoction					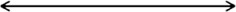				
placebo					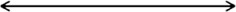				
Assessments:									
Physical examination	X	X	X	X	X	X	X	X	X
Vital signs	X	X	X	X	X	X	X	X	X
CARIFS scale	X	X	X	X	X	X	X	X	X
Child pneumonia syndrome combined efficacy evaluation scale	X	X	X	X	X	X	X	X	X
CABP symptoms/signs	X	X	X	X	X	X	X	X	X
Pulse oximeter	X	X	X	X	X	X	X	X	X
12-lead ECG	X							X	X
Chest X-ray	X							X	
Laboratory testing	X			X		X		X	
Research drug management								X	
Adverse events	X							X	X
Economic indicators									X

CARIFS = Canadian Acute Respiratory Illness and Flu Scale.

This multicenter clinical study will be implemented in 5 centers in China, including Longhua Hospital Affiliated to Shanghai University of Traditional Chinese Medicine; Dongfang Hospital of Beijing University of Chinese Medicine; Municipal Hospital of Traditional Chinese Medicine Affiliated with

Shanghai University of Traditional Chinese Medicine; Affiliated Hospital of Liaoning University of Traditional Chinese Medicine and Affiliated Hospital of Changchun University of Traditional Chinese Medicine.

### 2.2 Recruitment

To recruit participants, we will publicize by such means of recruitment posters, leaflets, and WeChat public platforms. During recruitment, we will fully communicate with the patient and their guardians, clearly informing them of the purpose, significance, test process, treatment methods, and possible benefits and risks of the study. We will then inform the patients and their guardians one-by-one of the contents of the informed consent form. When patients and their guardians completely agree with all the items in the consent form, they will sign it of their own free will.

### 2.3 Participants

Children with community-acquired pneumonia (CAP) will be identified on hospital admission by pediatricians. Those children who have bacterial infection on admission will be identified. Any patient who meets the inclusion criteria and the exclusion criteria will be recruited by providing written informed consent. The inclusion and exclusion criteria are listed in detail.

#### 2.3.1 Inclusion criteria

1. Male and female children aged less than 5 years and older than 1 year;
2. Children with CAP will meet the following criteria:
  - The imaging findings (chest X-ray or chest CT) are consistent with the characteristics of bacterial pneumonia, including new, or progressive infiltrating inflammation of the lung. Chest X-rays show punctate or patchy infiltration shadows of the pulmonary parenchyma, increased texture, blurred or striped shadows in both lungs, clustered reticular, or deepened shadows of the hilum, or even infiltration around the hilum. Chest CT shows patchy and nodular increased density shadows or pulmonary texture thickening and patchy increased density shadows.
    and
  - Acute course of disease (< 5 days);
    and
  - At least 3 clinical manifestations meet the following symptoms or signs of lower respiratory tract infection:
    a. New onset or aggravation of cough;
    b. Purulent sputum or a change in the nature of the sputum;
    c. Axillary temperature (>38.5°C);
    d. Dyspnea or shortness of breath or hypoxemia (SPO2 saturation in indoor air < 92%) (sea level) or < 90% (plateau);
    e. Lung auscultation consistent with pneumonia (i.e., rough or reduced breath sounds can be heard in the early phase, and coarsening or dry-wet rales or tubular breathing sounds can be heard in the late phase);
    f. White blood cell count > 10^9^/L, neutrophil percentage >50%;
    g. C-reactive protein >10 mg/L; h. PCT > 0.5 μg/L.
3. Meeting the TCM syndrome differentiation criteria of asthma, cough of pneumonia, and wind-heat stagnation of lung syndrome: Guidelines for the Diagnosis and Treatment of Common Diseases in Pediatrics of TCM (2012) by Chinese Academy of Traditional Chinese Medicine.^[15]^
  Main symptoms: fever; cough; asthma and nasal agitation; yellow phlegm or phlegm hoarseness in the throat. Secondary symptoms: nasal obstruction, runny or yellow discharge; redness of the pharynx; headache; reddish complexion; thirst; constipation; yellow urine. Tongue, pulse and fingerprint: red tongue; thin yellow fur; pulse floating and fast, fingerprint floating purple. Presence of at least 2 main symptoms and at least 2 secondary symptoms: the diagnosis can be made by referring to the tongue, pulse and fingerprints.
4. Children who can be admitted to the hospital for routine treatment;
5. The informed consent process conforms to the requirements, and the legal agent signs the informed consent form.

#### 2.3.2 Exclusion criteria

1. Pneumonia is identified or suspected to be caused by non-community-acquired bacterial pathogens (e.g., ventilator-associated pneumonia, hospital-acquired pneumonia, aspiration pneumonia, suspected viral, fungal, and partial *Mycobacterium pneumoniae* infections);
2. Noninfectious causes of infiltration (such as pulmonary embolism, chemical pneumonia caused by inhalation, hypersensitivity pneumonitis, congestive heart failure);
3. Pleural empyema (excluding nonsuppurative pneumonia pleural effusion);
4. Confirmed or suspected of infection with atypical pathogens based on the epidemiological background (*Mycoplasma pneumoniae* MP antibody titer >  = 1:160 or IgM positive; *Chlamydia pneumoniae* single serum IgM titer > = 1:16 or Ig G titer > = 1:512) virus (such as Coxsackie virus, influenza A virus, influenza B virus, human parainfluenza virus, human respiratory syncytial virus, adenovirus IgM positive) infection;
5. Including but not limited to pneumonia with bronchial asthma, bronchial foreign body, pneumonia with measles, whooping cough, influenza, pneumonia with other serious primary diseases of the lung;
6. CAP requiring the patient to enter the ICU for treatment;
7. 72 hours before enrollment: patient who used antibiotics (except for one-day oral or intravenous short-acting antibiotics; systemic antibacterial therapy ≥ 48-hour failure and isolation of previously used systemic antibiotic-resistant pathogenic microorganisms);
8. Oral administration according to a doctor's prescription within 72 hours before enrollment Chinese medicine, Chinese patent medicine treatment (excluding medication > = 48-hour failure);
9. Use of a traditional Chinese medicine injection within 72 hours before enrollment;
10. Use of the Chinese medicine administered in this test within 72 hours before enrollment (Maxing Shigan Tang and Qingfei experience prescription);
11. Use of ceftriaxone (or other third-generation cephalosporin antibiotics) to treat a CABP patient for whom previous treatment was ineffective on the basis of the clinical or epidemiological background or the isolation of ceftriaxone-resistant pathogenic microorganisms in the early stage of CABP;
12. Previous use of β-lactam antibiotics and allergens in the study of traditional Chinese medicine components;
13. Previous history of epilepsy or convulsions (except for well-documented children with febrile seizures);
14. Requirement, for any reason, of simultaneous antibacterial or systemic antifungal treatment except topical antifungal or antibacterial treatment, a single oral antibiotic treatment of vaginal candidiasis;
15. Patients with neoplastic lung disease, cystic fibrosis, fatal diseases, chronic neurological diseases affecting the clearance of lung secretions, or a life expectancy of less than or equal to 3 months;
16. Use of probenecid within 3 days before starting the study treatment plan or the necessity to be treated with probenecid;
17. Infection that may require the use of systemic corticosteroids simultaneously;
18. Evidence of immediate life-threatening illnesses, including but not limited to current or impending respiratory failure, acute heart failure, shock, acute liver failure, and active gastrointestinal bleeding;
19. Primary disease of the heart, liver, kidney, digestion and hematopoietic system;
  a. 12-lead ECG abnormalities, including but not limited to ST-T changes, pathological Q waves, various arrhythmias, such as sinus tachycardia, bradycardia, ventricular premature beats, atrial premature beats, atrial fibrillation, supraventricular and ventricular tachycardia, and various conduction blocking;
  b. AST, ALT, TBIL, ALP > upper limit of normal value (1.5 times);
  c. BUN, Cr > upper limit of normal value;
  d. platelet count <100 × 10^9^/L;
  e. neutropenia <1.5 × 10^9^/L.
20. Pneumonia with diarrhea;
21. Severe malnutrition, immunodeficiency, congenital airway malformation, abnormal lung development;
22. Participation in any drug or medical-device clinical trial within 30 days before enrollment or participation in this trial before;
23. Unable or unwilling to follow the procedures and restrictions specified by the study;
24. Any situation that the researcher believes will compromise patient safety or data quality.

### 2.4 Randomization and allocation concealment

Using the method of block randomization, with the center as the stratification factor, the layer will be divided into experiment 1 group, experiment 2 group, and simulation agent control group according to the ratio of 1:1:1. SAS statistical software will be used to generate a random number grouping table according to the case distribution and random proportion of 5 centers by the Institute of Basic Research in Clinical Medicine, China Academy of Chinese Medical Sciences (IBRCM). And central stochastic system of IBRCM will be used to perform the random allocation.

### 2.5 Blinding

The method of double blind and double simulation is adopted. The participants, outcome assessors, study coordinators, data managers, and statisticians will be blinded to the group allocation. There will be no difference in appearance, taste, smell, packaging, or label between the experimental drug and the placebo.

### 2.6 Interventions

In this clinical trial, the eligible patients will be randomly divided into 3 groups: experiment group 1, experiment group 2 and placebo control group which will be given Maxing Shigan Decoction, Qingfei Decoction or placebo respectively. The western medicine will be used in the groups as basic treatment. Western medicine treatment includes:

1. General treatment, including fluid therapy, if necessary, antipyretic treatment;

2. Antibiotic treatment: the antibiotic ceftriaxone sodium will be initially given 50 mg/kg/ time, once per day, with the total amount not exceeding 2 g per day, followed by intravenous drip treatment.

For patients with no significant relief or aggravation of clinical symptoms and signs at 72 hours after initial treatment, the antibiotic will be upgraded to cefepime 40 mg/kg/ time, one time /12 hours, and the single dose will be not more than 2 g.

Experiment group 1: Maxing Shigan Granule and Qingfei Yin simulation agent will be taken orally half an hour before meals for children aged 1 to 2 years old, 1 bag/time, 3 times a day. Children aged 3 to 5 years old, 2 bags/time, 3 times a day. The treatment course is 7 days.

Experiment group 2: Qingfei Yin Granule and Maxing Shigan simulation agent will be taken orally half an hour before meals for children aged 1 to 2 years old, 1 bag/time, 3 times a day. Children aged 3 to 5 years old, 2 bags/time, 3 times a day. The treatment course is 7 days.

Place control group: Maxing Shigan simulation agent and Qingfei Yin simulation agent will be taken orally half an hour before meals for children aged 1 to 2 years old, 1 bag/time, 3 times a day. Children aged 3 to 5 years old, 2 bags/time, 3 times a day. The treatment course is 7 days.

### 2.7 Outcomes

In this pilot study to evaluate the clinical efficacy of traditional Chinese medicine combined with antibiotics in the treatment of bacterial pneumonia in children, we aim to capture a wide range of clinically relevant endpoints that have been used in clinical trials of pneumonia in children and are considered by clinicians as important. These include the following:

#### 2.7.1 Primary outcome

1. Clinical cure rate: Defined as the time when the symptoms and signs of pneumonia disappeared completely or the symptoms and signs improved to no longer need further clinical treatment (Day 7, 14 ± 2 days).
2. Antibiotic application days: Calculated as the total days of antibiotic application from the first antibiotic administration to stable fever for at least 72 hours; or stable fever (axillary temperature <37.3°C) for at least 24 hours with significant improvement in the general symptoms; respiratory symptoms improved for 3 days (Day 7, 14 ± 2 days).

#### 2.7.2 Secondary outcomes

1. Complete antipyretic rate at the EOT (axillary temperature <37.3°C, and the above symptoms maintained for at least 24 hours); (Day 7)
2. Complete antipyretic days; (Day 7, 14 ± 2 days)
3. Achieved the rate of transfer to the ICU (indication)^[16]^ (Day 7, 14 ± 2 days)
4. Disease efficacy: It was divided into clinical recovery, clinical failure, and uncertain clinical efficacy.^[17]^ On Day 7 and Day 14 ± 2, clinical researchers will evaluate the disease efficacy of each case.
5. TCM syndrome efficacy: Refers to the guiding principles for clinical research of new Chinese medicine (Trial) (2002 Edition)^[18]^; (Day 7)
6. Effect time of antipyretic, cough, cough phlegm, antiasthmatic; (Days 0, 1, 2, 3, 4, 5, 6, 7, 14 ± 2 days)
7. Effect of a single symptom (fever, cough, cough, asthma) and pulmonary signs; (Days 0, 1, 2, 3, 4, 5, 6, 7, 14 ± 2 days)
8. Antibiotic upgrade treatment rates. (Day 7, 14 ± 2 days).

### 2.8 Adverse event reporting and treating

If participants experience adverse events (AE) during the trial, the investigators will record verified items, such as the date of onset, date of recovery, association with the investigational drugs, related measures and treatment, and registered in the adverse event record. When the adverse event occurs, the investigators can take necessary treatment measures according to the AE until it recovery or stabilization. If participants experience serious adverse events (SAEs) during the trial, the treatment method should be identified immediately. The patients will be suspended from the trial and treated as a lost case. Meanwhile, the clinical trial supervisor will be informed of the treatment results. The researchers should also register the SAEs. For safety assessment, blood, and urine tests will be conducted during the screening visit and the end of treatment.

### 2.9 Sample size

In the absence of existing data, this pilot study aims to recruit 240 participants over 21 months. A total of 240 participants will be randomly assigned to receive Maxing Shigan Decoction, Qingfei Decoction and placebo according to the proportion of 1:1:1 to evaluate the effect and safety of traditional Chinese medicine alone or combined with antibiotics on bacterial pneumonia in children. The results of this pilot study will inform the power calculation of future studies and determine the most appropriate endpoints of future trials. Past evidence and data suggest that we will be able to estimate a range of outcome parameters with a high enough degree of accuracy with a sample size of 80 and allow some initial comparisons between treatment and placebo. The number of patients included over this timeframe will inform the feasibility and design of further studies.

### 2.10 Data collection

The recruited participants will have their baseline data collected, including chest radiograph appearances, blood tests (complete blood count, PCT, CRP), and clinical observations. These parameters will be repeated throughout the participants hospitalization and upon discharge, as shown in Table 1. Participants adverse events, conditions requiring interventional management and other information will be noted in the case record form in detail. All participants will be asked to complete the evaluation scale of the curative effect of child pneumonia combined with the syndrome and the Canadian acute respiratory illness and flu scale (CARIFS) at their date of recruitment, at the time of hospitalization, at the time of hospital discharge and at the day of the follow-up visit. Additional blood tests and imaging will be performed at the discretion of the treating physicians.

All the above study data will be collected by clinical study staff using designated source documents or paper-based case report forms, which will then be entered into electronic databases. Clinical research data will be maintained through a combination of secure electronic data management systems and physical files with restricted access to ensure confidentiality. Data related to study endpoints will be extracted from the electronic databases for statistical analyses.

### 2.11 Data management

According to the case report form, the data management personnel of the Evaluation Center of IBRCM will establish a database system dedicated to this test before the study. Data collected will be stored in a database system that is dedicated to this test and has been established by the IBRCM. Primary data management activities include establishing a database, checking the data again before entry, data entry, data review, data locking and unblinding and data processing. The onsite study data manager oversees data-related procedures at the study site and is supervised by a data management staff that is unrelated to this study.

### 2.12 Data monitoring

For the trial to be adherent to clinical practice and for the trial data to be acknowledged upon registration both domestically and internationally, the clinical research associate (CRA) of the clinical research center of Changchun University of TCM will visit each clinical trial center regularly or according to the actual situation to conduct clinical trial supervision. In the process of monitoring, the CRA shall confirm whether all patients have signed the informed consent form, understand the inclusion rate of patients and the progress of the test, and check whether the records and reports of all test-related data are correct and complete. The records in the confirmation case report shall be consistent with the original data; all errors or omissions will be corrected or noted; the concomitant medication, concomitant diseases, lost visits, and examination omissions of each patient shall be confirmed and recorded; the withdrawal and lost visits of the selected patients shall be verified; all adverse events shall be recorded; serious adverse events shall be reported and recorded within the specified time record; and whether the test drug is supplied, stored, distributed and recycled according to relevant regulations and requirements will be verified and the corresponding records created.

### 2.13 Statistical methods

Statistical analysis will be performed using SAS version 9.4 or above. The measurement data will be compared between groups by independent sample *t* test. The comparison between the counting data sets will be performed by X^2^ test. Mann–Whitney nonparametric rank sum test will be used to compare the rank data sets. All clinical end points will be analyzed by intentionality analysis (ITT) and per protocol set (PP) analysis, and safety analysis will be performed by safety data set (SS).

The efficacy scores of patients with bacterial pneumonia will be calculated on the day enrolled and day 3 to day 5, and the mean, standard deviation, median, P5, P75, maximum, and minimum values of the difference between the treatment period and the baseline will be used to describe them. PP and ITT will be adopted to analyze the primary indicator and global indicators. Since this study is a multicenter, randomized, double-blind control trial, the effect of center effect on the outcome indicators should be considered in the analysis.

## 3 Discussion

CAP is a common and frequently occurring disease in childhood. More than 2 million children under the age of 5 die of pneumonia every year worldwide, accounting for 20% of the total dead population.^[19]^ Bacteria are the main pathogens causing CAP in children, and antibiotics are the selected treatment. However, in recent years, the emergence and spread of antibiotic resistance worldwide have threatened our ability to treat bacterial infectious diseases. Therefore, in this postantibiotic era, human beings urgently need an alternative to antibiotic therapy to address bacterial infectious diseases.

Traditional Chinese medicine has a unique theoretical system and rich clinical experience in the treatment of bacterial infectious diseases. The classical prescription “Maxing Shigan Decoction” and Professor Wang Lie “Qingfei Decoction” have significant curative effects in the treatment of childrens CABP,^[20]^ but their clinical efficacy and safety have not been systematically evaluated by large sample multicenter randomized controlled trials.

Therefore, this clinical trial is the first to evaluate the efficacy and safety of “Maxing Shigan Decoction” and “Qingfei Decoction” in the treatment of children with CABP. This clinical trial will obtain a wide range of clinically relevant endpoints to explore the potential role of the above drugs in the treatment of pediatric CABP. The research results will provide a reference for future research design.

## Author contributions

**Conceptualization:** Liping Sun.

**Data curation:** Lina Wei, Caiwen Wang, Xuejiao Wang, Haihang Sun, Lei Wang, Yijie Wang.

**Funding acquisition:** Liping Sun.

**Methodology:** Yutong Fei, Lin Luo.

**Project administration:** Yina Guo, Liping Sun.

**Resources:** Lina Wei, Liping Sun.

**Writing – original draft:** Lina Wei, Liang Liu, Jing Han, Xiaoting Ren, Bo Yao.

**Writing – review & editing:** Yina Guo, Liping Sun.
